# SURVEY ON STROKE REHABILITATION IN EUROPE (SUSTAIN-EU) TO EVALUATE EUROPEAN STROKE REHABILITATION SERVICES

**DOI:** 10.2340/jrm.v58.44888

**Published:** 2026-08-17

**Authors:** Guna BĒRZIŅA, Katharina S. SUNNERHAGEN, Ayse A. KÜÇÜKDEVECI, Mauro ZAMPOLINI, Volodymyr GOLYK, Yvona ANGEROVA, Marco FRANCESCHINI, Daiana POPA, Catarina AGUIAR BRANCO, Markos SGANTZOS, Paolo BOLDRINI, Dominic PERENNOU, Thierry LEJEUNE, Xiaolei HU

**Affiliations:** 1 Department of Rehabilitation, Riga Stradiņš University, Riga, Latvia; 2 Rehabilitation Medicine, Clinical Neuroscience, University of Gothenburg, Gothenburg, Sweden; 3 Rehabilitation Medicine, Sahlgrenska University Hospital, Gothenburg, Sweden; 4 Ankara University, Faculty of Medicine, Department of Physical Medicine and Rehabilitation, Ankara, Turkey; 5 Department of Rehabilitation, USL UMBRIA 2, Foligno, Italy; 6 Disability and Rehabilitation, Rehabilitation and Disability Unit, WHO Country Office in Ukraine; 7 Ukrainian Society of Physical and Rehabilitation Medicine, Dnipro, Ukraine; 8 Department of Rehabilitation Medicine First Faculty of Medicine, Charles University and General University Hospital in Prague, Czech Republic; 9 Research Area in Neuromotor Rehabilitation and Rehabilitation Robotics, Department of Neuroscience, IRCCS San Raffaele Roma, Rome, Italy; 10 Rehabilitation Hospital Felix Spa, Oradea, Romania; 11 PRM Service – Hospital Feira – ULSEDV, Portugal and FMD University of Porto, Portugal; 12 Medical School, University of Thessaly, Greece; 13 Private Practice, Ferrara, Italy; 14 Neurorehabilitation Department, Institute of Rehabilitation, Grenoble-Alpes University Hospital, Echirolles, France; 15 Lab Psychology and NeuroCognition, University of Grenoble-Alpes, Grenoble, France; 16 UCLouvain, Institut de Recherche Expérimentale et Clinique (IREC), Neuro Musculo Skeletal Lab (NMSK), Brussels, Belgium; 17 Cliniques universitaires Saint-Luc, Service de médecine physique et réadaptation, Brussels, Belgium; 18 Department of Community Medicine and Rehabilitation, Rehabilitation Medicine, Umeå University, Umeå, Sweden

**Keywords:** Europe, health services, stroke rehabilitation

## Abstract

**Objective:**

This study aimed to assess the organization and delivery of post-stroke rehabilitation services across Europe, focusing on the role of Physical and Rehabilitation Medicine physicians.

**Design and Methods:**

A cross-sectional online survey was conducted by the ESPRM Working Group of Stroke Rehabilitation from 30 European countries. The survey followed the International Classification of Service Organization in Rehabilitation framework and covered stroke care levels, rehabilitation services, registries, and quality control. Descriptive statistics were used for analysis.

**Results:**

With an 81% response rate, the study revealed both commonalities and disparities in service provision in 30 European countries. Stroke unit rehabilitation was available in 90% of countries, and inpatient services in 100%. However, early supported discharge (53%), community-based rehabilitation (63%), and telerehabilitation (57%) were less common. Countries joining ESPRM after 1991 had significantly lower rates of early supported discharge (−48%, *p*=0.01) and community-based care (−55%, *p*=0.02).

**Conclusion:**

Despite widespread inpatient services, major gaps remain in community- and technology- supported rehabilitation. Addressing these disparities requires targeted policy changes, altered resource allocation, and enhanced training to ensure equitable and effective stroke recovery across Europe.

## LAY ABSTRACT

Stroke is a major health issue in Europe, and many people need rehabilitation to recover and regain independence. However, the way rehabilitation services are organized and provided after a stroke varies widely between countries. To better understand this, we asked experts from 30 European countries about the services available in their regions. Most countries offer hospital-based rehabilitation, but many lack support for recovery at home or in the community. Services like home visits, online therapy, and help after leaving the hospital are not available everywhere. Countries that joined the European Rehabilitation Society more recently tend to have fewer of these services. This means that stroke survivors in some places may not get the help they need to recover fully. Our study highlights the need for better planning, training, and funding to make sure everyone in Europe has equal access to high-quality stroke rehabilitation, no matter where they live.

Stroke is the leading cause of disability with various long-term impairments, limitations of daily activities, restrictions of social participation and decreased quality of life among adults worldwide (1, 2). In the WHO European Region, stroke accounts for more than 1.5 million new events per year, with considerable variability in incidence and case fatality between Western, Central, and Eastern Europe (3, 4). Within the European Union specifically, stroke incidence has been estimated at 1.12 million per year, with 9.53 million stroke survivors, a figure projected to grow by 27% between 2017 and 2047, particularly in Eastern Europe (5). This has resulted from a growing elderly population, in conjunction with a decreased acute stroke mortality. The growing number of stroke survivors with various post-stroke disabilities not only leaves the survivors suffering after the stroke but also creates a heavy social and financial burden on their families and society. Rehabilitation therefore plays a pivotal role in maximizing functional recovery and promoting independence in activities of daily living and social participation and reducing the long-term burden on individuals, caregivers, and healthcare systems.

The responsibility of the multi-professional interdisciplinary rehabilitation team is to provide services in a coordinated and goal-oriented manner, to facilitate the person’s return to daily life as early as possible (6, 7). Effective rehabilitation interventions after stroke can improve recovery and reduce disability. This, in turn, can improve the quality of life of persons with stroke and their families and mitigate significant and long-term costs. In rehabilitation research, 3 levels of analysis are commonly distinguished: the micro level (individual patient–professional interaction and single interventions), the meso level (organization of the service chain across institutions and settings of care), and the macro level (national policies and health-system regulation) (8, 9). Most rehabilitation studies have focused on delivering various interventions targeting specific rehabilitation needs of individuals, which relates more to the micro level of care (8), i.e., they have not considered the impact on the organization. In contrast, the meso level of care regarding organization of the rehabilitation service chain from hospital to local community has been less studied (10). Post-stroke rehabilitation is often fragmented even though the continuum of rehabilitation is highly needed among a huge number of persons after stroke. There are some empirical considerations on the wide variation of rehabilitation services in Europe due to the vast cultural and economic diversity not only among the countries but also within the country (4), considering health management and various health systems (macro level). Stroke care overall in Europe is fragmented and needs improvement, as noted in the “Action Plan for Stroke in Europe”, where rehabilitation and life after stroke are part of the plan and considered to be under-prioritized. Still, it remains largely unknown how much stroke rehabilitation services differ within European countries and regions.

While rehabilitation services were slowly but purposefully developed in Western Europe after the Second World War, the collapse of the Soviet Union marked an absolutely new era in Eastern Europe, including in the field of rehabilitation. This naturally led to the establishment of new professions and services in rehabilitation in these countries and their successive entry into the European Society of Physical and Rehabilitation Medicine (ESPRM). Thus, from the 5 countries at the founding phase, ESPRM had grown to 20 countries in 2004 (11) and 37 countries in 2024.

The ESPRM (https://esprm.eu/) Working Group (WG) of stroke rehabilitation (ESPRM-WG Stroke rehabilitation) is the primary scientific unit and the driving force in ESPRM to promote the role of PRM in interdisciplinary stroke rehabilitation and to provide clinical information regarding stroke rehabilitation services in European countries. ESPRM-WG Stroke rehabilitation aims to promote evidence-based clinical practice, education, and research and by connecting PRM physicians with common interests in stroke rehabilitation.

Mapping the differences in stroke rehabilitation services within Europe will provide important information for better planning and organization of stroke rehabilitation services through inter-regional and national comparisons. Recently, an International Classification of Service Organization in Rehabilitation (ISCO-R) (9) has been constructed to describe the meso-level of healthcare in a standardized way. By using ISCO-R as a framework, ESPRM-WG Stroke rehabilitation developed a survey regarding stroke rehabilitation services.

The overall aim of the present study is to provide an overview of post-stroke rehabilitation services, highlighting the current status of their organization and delivery in European countries, including the role of PRM physician.

## METHODS

### Study design and management

This cross-sectional study was carried out by ESPRM-WG Stroke rehabilitation during 2023–2025.

The members of ESPRM-WG Stroke rehabilitation (co-authors) developed a survey through a multistage consensus process and conducted a pilot to avoid potential process problems.

National delegates from all 37 ESPRM member countries were invited to participate in the study. The online survey form was created using Google Forms. The survey link, the invitation to participate, and a PDF version of the survey form were sent to all national delegates of the ESPRM in July 2023. Several reminders to fill in the survey were sent out every 2 months and the survey was closed in April 2024.

Depending on the organizational situation within each country, the delegate or delegate’s nominated representative of the country was responsible for answering the survey regarding the organization of stroke rehabilitation on either the national level or the regional level depending on the considerable variations between countries and regions in the same country.

### Survey

The survey was based on the framework of the ISCO-R (9), including contents from the Road Map for Quality Stroke Care, published by the World Stroke Organization (18). The survey was named SUSTAIN-EU – Survey on Stroke Rehabilitation in Europe.

After 2 feedback rounds within ESPRM-WG Stroke rehabilitation, the survey was presented for discussion and approval at the ESPRM General Assembly and UEMS-PRM Section and Board meeting*.*

The SUSTAIN-EU survey (Appendix S1) includes information on: the individual completing the form and providing country information, situation on PRM physicians in the country, questions related to stroke epidemiology, general information on the organization of stroke rehabilitation, use of telerehabilitation for stroke patients, and stroke rehabilitation registries and quality control. Definitions of the services used for the survey are given in Table I.

**Table I:** Definitions of stroke services

Item	Service	Definition
Acute services	Stroke unit	Organized stroke unit care is a form of care provided in hospital by nurses, doctors, and therapists who specialize in looking after stroke patients and work as a coordinated team (24). Organized inpatient (stroke unit) care is a term used to describe the focusing of care for stroke patients in hospital under a multidisciplinary team who specialize in stroke management (25)
	Rehabilitation for stroke patients in acute wards	Establishment of a mobile visiting PRM team under the responsibility of a PRM physician, while the patient remains in the referring specialist’s care (acute rehabilitation team (ART). Daily visits to the acute wards by specialists from a standalone PRM facility. Establishment of facilities in PRM centres to take patients very early to start their PRM programme (26)
	Early supported discharge	Team offering rehabilitation in the community that replicates the stroke unit care; this enables earlier home discharge than would be possible if the team was not available (27) (reach out or reach in)
Post-acute services	Inpatient rehabilitation	Goal-oriented multi-professional rehabilitation under the responsibility of a PRM physician in the inpatient rehabilitation facility (28)
	Day rehabilitation	Goal-oriented multi-professional rehabilitation under the responsibility of a PRM physician in the outpatient rehabilitation facility (29)
	Outpatient – ambulatory rehabilitation	Single rehabilitation services in the outpatient rehabilitation facility (29)
	Home rehabilitation	Rehabilitation at home to replace institutional rehabilitation (30)
	Home based rehabilitation	Home exercise to prevent deterioration and promote health through physical activity (30)
Long-term services	Community-based rehabilitation	Strategy within general community development for the rehabilitation, equalization of opportunities, poverty reduction, and social inclusion of people with disabilities (31)
	Follow-up or intermittent rehabilitation	Intermittent inpatient rehabilitation services can be used to induce and bolster rehabilitation effects in patients with chronic health conditions, in particular if they are related to psychosocial stress and vocational problems
Other forms	Telerehabilitation	Delivery of rehabilitation services via information and communication technologies (32)
	Other specified forms of rehabilitation	If the organized rehabilitation service does not fall under any of the previously listed definitions

Overall information on stroke care standards includes having an established/defined programme, plan, or strategy; a stroke rehabilitation pathway; a pay-for-performance strategy; and stratification for health service departments.

The variables for telerehabilitation were assessment/evaluation of patient, diagnostics, setting goals, treatment/rehabilitation therapy, education, and monitoring/follow-up. The types of telerehabilitation were 2-way real-time visits with audio and/or video, asynchronous e-visits, virtual check-ins, remote evaluations of recorded videos or images, telephone assessment, and management services. The purposes and types were reported if they were used for at least 1 rehabilitation service. Overall proportions of purposes of telerehabilitation were reported.

Information was collected regarding the use of National stroke registries, coverage of the stroke population, and whether they are used for reporting data on rehabilitation, as well as the existence of measures regarding quality control (reported as used if used for at least 1 rehabilitation service).

### Data presentation and analyses

Data were downloaded from Google Forms and processed in a Microsoft 365 Excel (Microsoft Corp, Redmond, WA, USA) spreadsheet. Descriptive statistics (frequencies) were calculated for all variables.

If multiple answers were provided for the same country, the service was considered present if at least 1 participant mentioned it. In order to provide uniform information on the overall population of countries, data from World Population Prospects 2024 from the Department of Economic and Social Affairs Population Division of the United Nations were used (12). Map Chart was used to illustrate the coverage of participating European countries (13).

To demonstrate the heterogeneity on the availability of various rehabilitation services clearly, we divided the participating countries into 2 groups based on the time of joining the ESPRM with a cut-off at 1991. This cut-off was based on historical facts on development of the ESPRM, as the majority of the participating countries in the study belong to the high-income countries according to the World Bank (14).

Comparison between groups was calculated using Fisher’s exact test by IBM SPSS Statistics version 29.0 (IBM Corp, Armonk, NY, USA). The following information was analysed: (*i*) characteristics of the participating countries and their representatives; (*ii*) situation of PRM physicians in each country; (*iii*) stroke epidemiology; (*iv*) stroke rehabilitation organization; (*v*) use and purpose of telerehabilitation services; (*vi*) stroke registries and quality control.

### Data availability

The data associated with the paper are not publicly available but are available from the corresponding author on reasonable request.

## RESULTS

### Characteristics of the participating countries and representatives of the countries

**Fig. 1:**
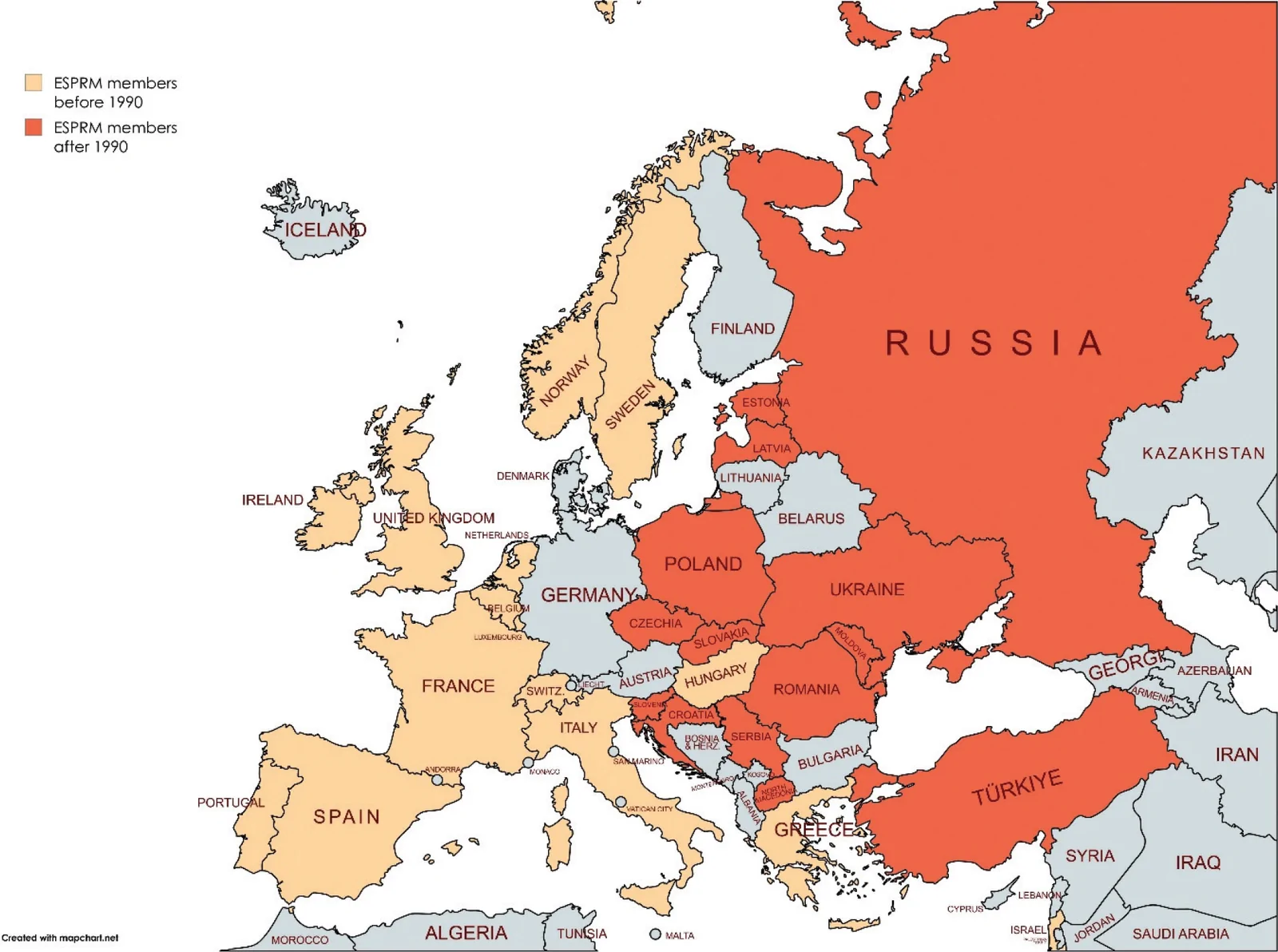
Choropleth map showing participation of countries in the SUSTAIN-EU survey and when they joined ESPRM.

The overall response rate for the survey was 81% with 30 of 37 countries completing the study. Six countries did not respond despite multiple contact attempts. Fourteen participating countries joined the ESPRM before 1991 while 16 participating countries joined after 1991 (Fig. 1). Denmark was excluded from the survey because the PRM is not recognized as an independent medical specialty there (15) and therefore no information on stroke rehabilitation was provided.

Thirty-five representatives (all PRM physicians) from 30 countries completed the survey, of whom 26 are ESPRM National Delegates. Three are individual members and 6 do not hold any position within the ESPRM. Three individuals were additionally certified in neurology, 2 in cardiology, and 1 in public health. There were 18 females and 17 males among the representatives.

### Description of overall population and stroke epidemiology in participating countries

The overall populations of participating countries are presented in Appendix S1.

Participating countries, when asked to provide information regarding stroke epidemiology, most commonly as data sources reported estimates (18 countries) and data from registries (11 countries). Only 1 country (Croatia) reported gathering information from scientific reports. As a source of information, Italy mentioned government documents and Ukraine listed data from e-Health and Public Health Centre. All countries, except the Netherlands and North Macedonia, reported data on the estimated annual number of incident strokes (N/100 000 inhabitants). However, reported data were highly inconsistent with available stroke incidence statistics provided by the Burden of Stroke in Europe Report and data from the European Society of Cardiology (3, 16).

### Situation of PRM physicians within the countries and their involvement in post-stroke rehabilitation

**Fig. 2:**
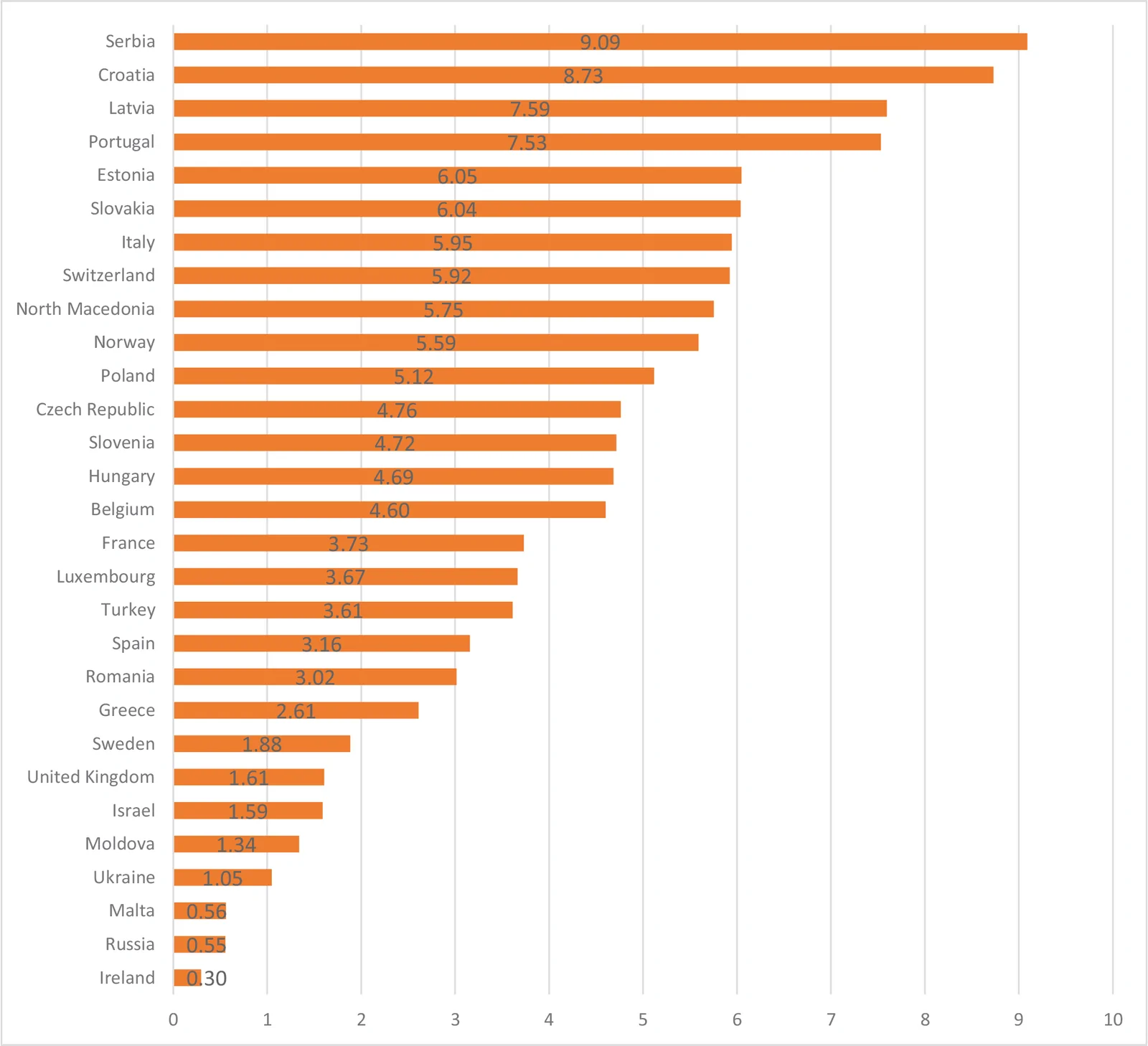
Proportion of PRM physicians per 100,000 inhabitants. (n = 29; data not available for the Netherlands)

**Fig. 3:**
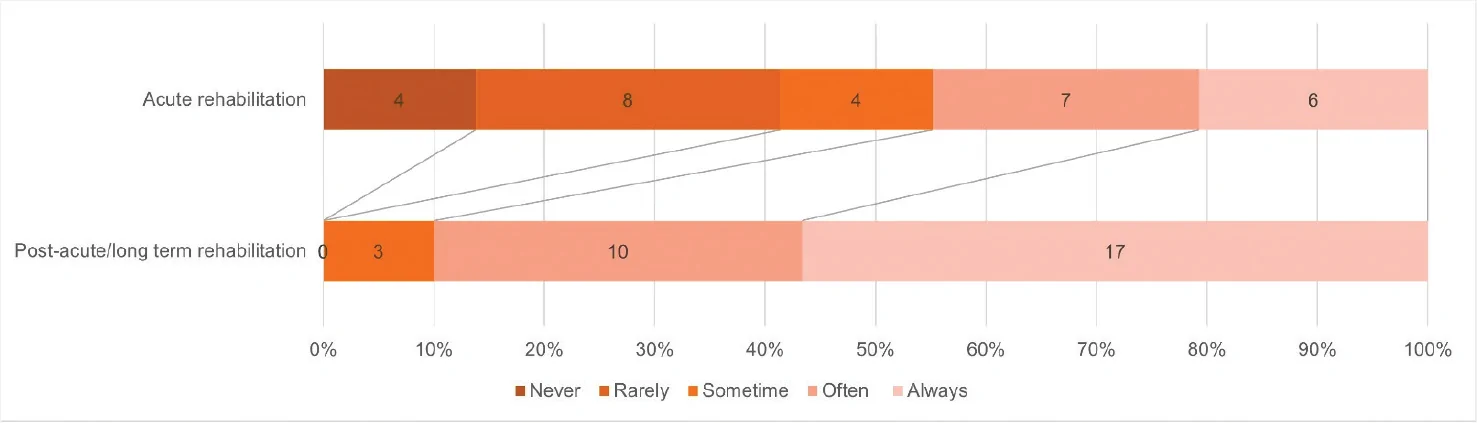
Distribution of PRM physicians officially responsible for stroke rehabilitation in the study countries.

Reported numbers of PRM physicians per 100,000 inhabitants in each of the countries vary between 0.3 in Ireland and 9.1 in Serbia, with a median of 4.2 (IQR 1.8–5.2) (Fig. 2). Four countries report that PRM physicians are never officially responsible for the acute rehabilitation of stroke patients (Moldova, Israel, Greece, North Macedonia), and only 6 countries are always involved of PRM physicians in acute stroke rehabilitation (United Kingdom, Italy, Estonia, Portugal, Serbia, and the Czech Republic). However, in almost all countries except 3 (Ukraine, the Netherlands, and Switzerland), PRM physicians are always or often involved in post-acute and long-term rehabilitation services for stroke patients (Fig. 3).

### Stroke care across countries

Twenty-seven countries reported having a programme, plan, or strategy for stroke rehabilitation. Among them, 25 operate nationally, 6 regionally, and 6 locally. A stroke rehabilitation pathway is established in 24 countries. Five countries follow the World Health Organization’s guidelines, 12 the European Stroke Organization’s, 22 the National guidelines, and 1 country uses both AHA/ASA and Canadian guidelines. Only 9 countries reported using a “pay for performance” strategy in stroke rehabilitation. Twenty-four countries have stratification of stroke service departments (stroke unit, stroke centre, comprehensive stroke unit) according to the Action Plan for Stroke in Europe 2018–2030 (4). Stroke care standards as reported by countries are presented in Table II.

**Table II:** Stroke care standards reported for each participating country

Country	Established/defined programme/plan/strategy	Stroke rehabilitation pathway	Pay-for-performance strategy	Stratification of health service departments
Belgium	-	-	-	+
Croatia	+	+	-	+
Czech Republic	+	+	+	+
Estonia	+	+	+	+
France	+	+	+	+
Greece	+	-	-	+
Hungary	+	+	-	+
Ireland	+	+	-	-
Israel	+	+	-	+
Italy	+	+	+	+
Latvia	+	+	-	-
Luxembourg	-	-	-	+
Malta	+	+	-	-
Moldova	+	-	+	+
Netherlands	+	+	-	+
North Macedonia	+	+	-	-
Norway	+	+	-	+
Poland	+	+	-	+
Portugal	+	+	+	+
Romania	+	-	-	-
Russia	+	+	+	+
Serbia	+	+	+	+
Slovakia	+	+	-	+
Slovenia	+	+	-	+
Spain	+	+	-	+
Sweden	+	+	-	+
Switzerland	+	+	+	+
Turkey	+	-	-	+
Ukraine	-	+	-	-
United Kingdom	+	+	-	+
Total	27	24	9	24

+ Stroke care standards reported by the country; - Stroke care standards *not* reported by the country. *ESPRM members before 1991 are not highlighted (white background) and members after 1991 are highlighted with a grey background.

### Stroke care pathway

In acute services, rehabilitation demonstrated a more homogeneous picture in the stroke unit (27/30) and rehabilitation for stroke patients in acute wards (24/30). However, only half (16/30) of the countries provide early supported discharge service (Table III), one of the least reported services. In post-acute care, inpatient rehabilitation (30/30) and outpatient/ambulatory rehabilitation services (29/30) were reported in almost all participating countries, while day (25/30) and home (20/30) rehabilitation services demonstrated more variations. Long-term services showed substantial heterogeneity in all 3 services, which are community-based rehabilitation (19/30), follow-up/intermittent rehabilitation (21/30), and telerehabilitation (17/30).

**Table III:** Available rehabilitation services for each participating country

Item	Acute services			Post-acute services				Long-term services		
Country	Rehabilitation in stroke unit	Rehabilitation in acute ward	Early supported discharge	Inpatient rehabilitation	Day rehabilitation	Home rehabilitation	Outpatient/ambulatory rehabilitation	Community-based rehabilitation	Follow-up/intermittent rehabilitation	Telerehabilitation
Belgium	+			+	+	+	+	+		+
Croatia	+	+	+	+		+	+		+	
Czech Republic	+	+		+	+		+	+	+	
Estonia	+	+		+	+	+	+	+	+	+
France	+	+	+	+	+		+	+	+	+
Greece	+	+	+	+	+	+	+	+		+
Hungary	+	+		+	+	+	+	+	+	
Ireland	+	+	+	+	+	+	+	+	+	+
Israel		+	+	+	+	+	+	+	+	+
Italy	+	+	+	+	+	+	+			+
Latvia	+	+		+	+	+	+		+	
Luxembourg	+	+	+	+	+	+	+	+	+	
Malta	+	+	+	+	+		+	+	+	
Moldova	+			+			+			
Netherlands	+	+		+	+	+	+	+	+	+
North Macedonia				+		+	+			
Norway	+		+	+	+	+	+	+	+	+
Poland	+			+	+	+	+			
Portugal	+	+	+	+	+	+	+	+	+	+
Romania		+		+	+		+			
Russia	+	+		+	+					+
Serbia	+	+	+	+			+		+	
Slovakia	+	+	+	+	+	+	+	+	+	
Slovenia	+	+		+	+		+	+	+	+
Spain	+	+	+	+	+	+	+	+	+	+
Sweden	+	+	+	+	+	+	+	+	+	+
Switzerland	+	+	+	+	+		+	+	+	
Turkey	+	+		+	+	+	+		+	+
Ukraine	+			+			+			
United Kingdom	+	+	+	+	+	+	+	+	+	+
Total	27	24	16	30	25	20	29	19	21	16

+ Rehabilitation service reported as available in country. *ESPRM members before 1991 are not highlighted (white background) and members after 1991 are highlighted with a grey background.

Moreover, 17 out of 30 countries reported that the stroke care between regions did not differ, 11 countries reported differences between regions, and no information was available regarding this topic for Ukraine and Moldova. Interestingly, even in countries where the organization of stroke care – including rehabilitation – is defined at the national level and does not formally differ between regions, respondents still reported differences between rural and urban areas in terms of the availability of rehabilitation services, and an overall limited number of beds dedicated specifically to rehabilitation.

Additionally, 1 country reported vocational rehabilitation as part of stroke care under “other rehabilitation services”. There were 5 countries (Ireland, Portugal, Spain, Sweden, and the United Kingdom) that reported having all defined rehabilitation services.

**Fig. 4:**
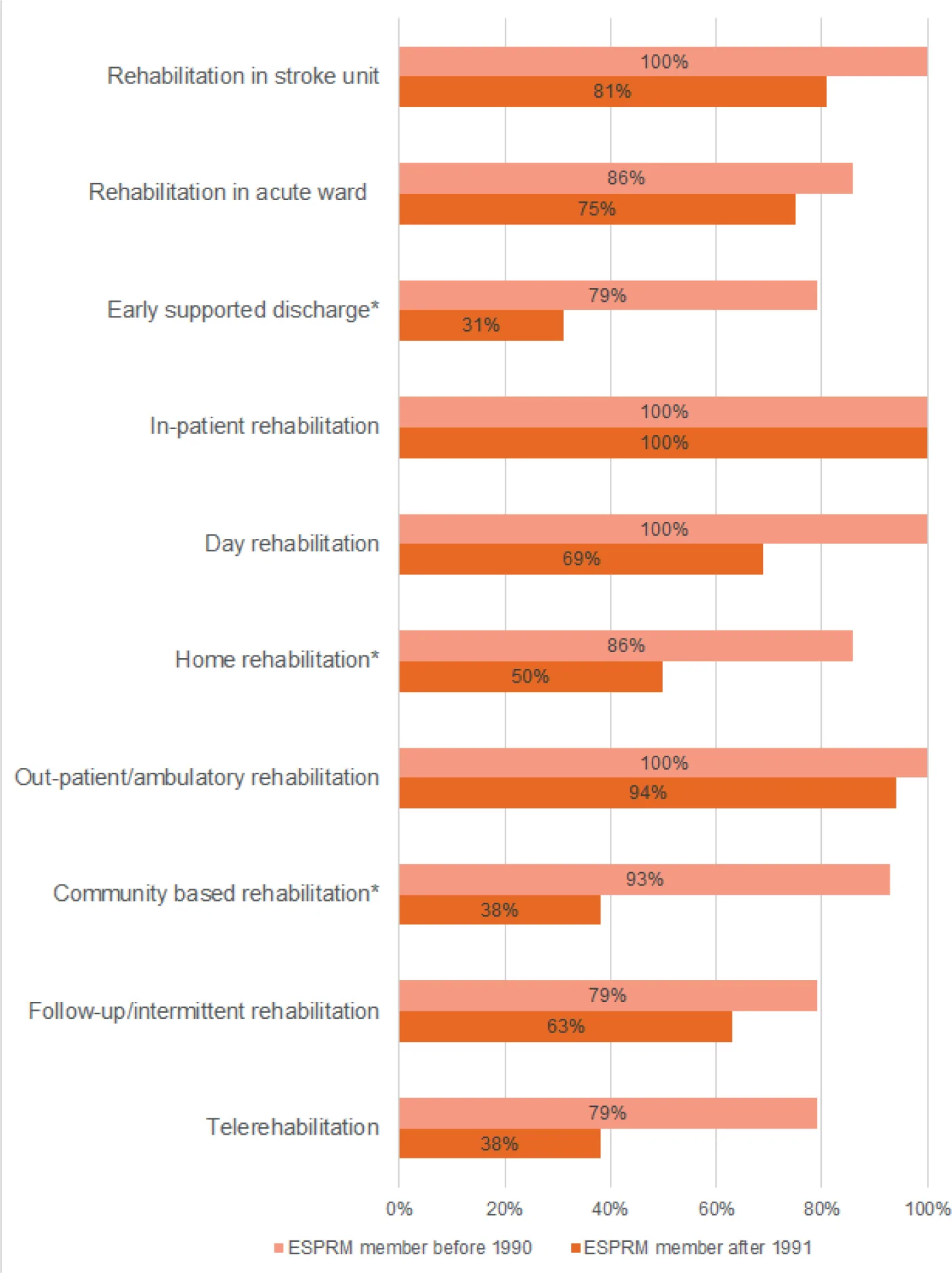
Proportions of rehabilitation services between old and new members of the ESPRM.

Differences in stroke rehabilitation services between countries that were members of the ESPRM before and after 1991 are shown in Fig. 4. Fisher’s exact test showed statistically significantly fewer services in early supported discharge (48% less, *p* = 0.01), home rehabilitation (36% less, *p* = 0.04), and community-based rehabilitation (55% less, *p* = 0.02) in the new countries of the ESPRM compared with the old countries. Even though the differences in rehabilitation in stroke unit, day rehabilitation, and telerehabilitation were 19%, 31%, and 41%, respectively, the differences were not statistically significant. Great similarities were found in rehabilitation in the acute ward, inpatient, outpatient/ambulatory rehabilitation, and follow-up/intermittent rehabilitation.

### Regional differences

The Netherlands, Italy, Portugal, Spain, and Switzerland reported regional differences due to municipal policies governing the organization of rehabilitation services. This leads to differences in service pathways, timing, standards, duration, connection to social assistance, access to technical aids, and other rehabilitation-related services between regions.

### Telerehabilitation

Only 16 countries reported telerehabilitation as part of stroke care. However, when analysing telerehabilitation within services, only 6 countries reported no remote rehabilitation (Croatia, Luxembourg, Poland, Romania, Serbia, and Slovakia).

**Fig. 5:**
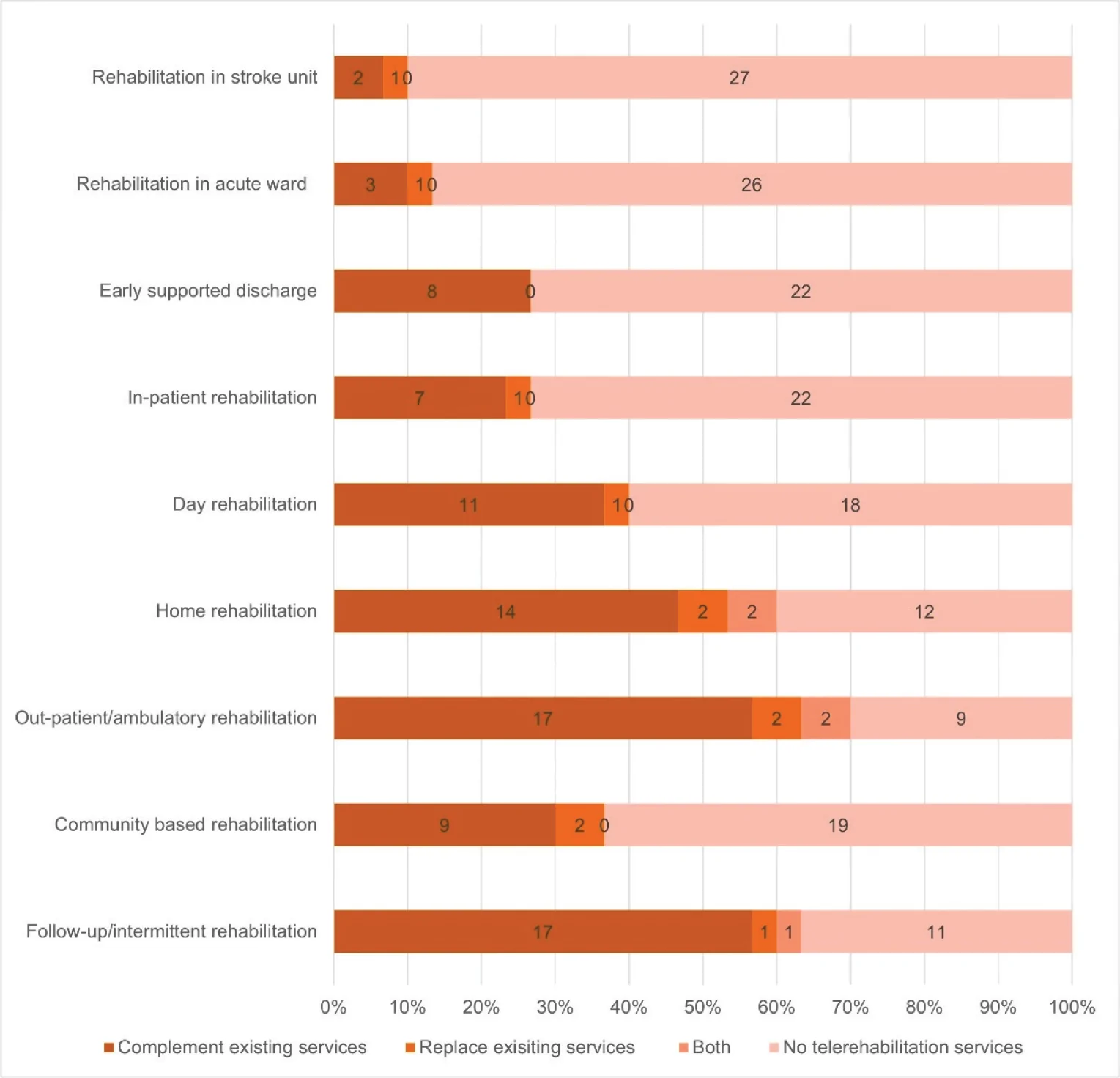
Percentage distribution of use of telerehabilitation in various stroke rehabilitation services in the study countries.

Telerehabilitation services showed broad variation across European countries, in terms of both purpose (assessment, goal setting, treatment, education, monitoring) and modality (2-way real-time audio/video, asynchronous e-visits, remote evaluation of recordings, telephone-based services). Most telerehabilitation services are implemented with the primary aim of complementing existing in‑person rehabilitation pathways. In these cases, digital tools are used to extend the reach of traditional care by providing additional monitoring, follow‑up, patient education, or low‑intensity exercise sessions between face‑to‑face appointments. In contrast, only a limited number of telerehabilitation interventions are designed as full substitutes for conventional services. Proportionately, most services are offered to outpatients in the post-acute or chronic phase after stroke (Fig. 5). Telerehabilitation services were mainly used for treatment/rehabilitation therapy (16 countries) and education (16 countries), followed by monitoring/follow-up (14 countries). In 10 countries, telerehabilitation was used for the assessment/evaluation of patients, and in 5 it was used for diagnostics. In 5 countries, all forms of telerehabilitation services were used. The most common methods were 2-way real-time audio/video visits (19 countries) and telephone assessments (18 countries), followed by virtual check-ins (13 countries) and remote evaluations of recorded videos or images (12 countries). Asynchronous e-visits were used in only 6 countries, making it the least utilized telerehabilitation service.

### Stroke rehabilitation registries and quality control

Sixteen out of 30 countries reported that they had National stroke registries. Four of these countries had no information on the proportion of stroke patients covered by the registry, and median coverage for these countries was 90% (IQR=78–100%), with the highest coverage reported for Hungary, Slovakia, and Slovenia (100%) and the lowest for Spain (40%). The stroke registry contains information regarding rehabilitation: (*i*) in stroke unit, 8 countries; (*ii*) in the acute ward, 6 countries; (*iii*) early supported discharge, 3 countries; (*iv*) inpatient rehabilitation, 6 countries; (*v*) day rehabilitation, 4 countries. In 5 countries, reported data in the stroke registry were used to identify problems or shortcomings in service delivery, and in 4 countries to design activities to overcome these deficiencies. Also reported were follow-up monitoring to ensure the effectiveness of corrective steps, accreditation certification procedures, organized audits, an appointed quality manager, and single intervention explicitly to improve structure, process, or outcome quality. Each of those items was reported by 4 countries.

## DISCUSSION

This cross-sectional survey study provides an overview of the current status of post-stroke rehabilitation services in European countries. With a response rate of 81%, the study revealed both similarities and heterogeneity in the organization and delivery of stroke rehabilitation services across the 30 participating European countries. Similarities were found in the availability of rehabilitation services in stroke units, in- and outpatient services, and telerehabilitation. Substantial differences were observed in the availability of early supported discharge, home and community-based rehabilitation services, the number and the involvement of Physical and Rehabilitation Medicine (PRM) physicians, telerehabilitation, and utilization of stroke registries and quality control measures. Many participating countries joined the ESPRM later than 1991 and this was associated with the differences in stroke rehabilitation services observed in the study. There were even regional differences within some countries.

Rehabilitation is crucial to all healthcare processes, including post-stroke care (17, 18). We found that the most common rehabilitation services were rehabilitation in the stroke unit in the acute service and in- and outpatient rehabilitation in the post-acute service in almost all participating European countries. These similarities may possibly reflect general acceptance of the pivotal role of rehabilitation for improving patients’ functioning and independence across Europe. However, it has been observed that there are significant gaps in the accessibility and quality of these rehabilitation services for stroke worldwide (19). Stroke can result in various impairments, activity limitations, and participation restrictions that can be addressed through services involving single or multiple professionals and interventions (20). The field of PRM is responsible for ensuring optimal outcomes and efficient patient navigation through the healthcare system and resource utilization. Our findings should be read in the light of the UEMS-PRM framework for rehabilitation, according to which effective rehabilitation must be evidence-based, timely, needs-based, multiprofessional, functioning-oriented, defined within an Individual Rehabilitation Project, continuous, and integrated within the overall healthcare process (6, 7, 20).

There was substantial heterogeneity regarding early supported discharge and long-term services. The countries joining ESPRM after 1991 reported significantly less provision in early supported discharge and home rehabilitation followed by community-based rehabilitation, compared with the countries joining ESPRM before 1991. As early supported discharges have shown strong evidence for shortening length of stay and improved independence (21), this type of service should be established across Europe. Besides the differences between countries, many countries reported regional variations. The significant disparities observed in the availability of various rehabilitation services across different regions underscore the need for a deeper understanding of the underlying factors. These differences may be attributed to variations in healthcare policies, resource allocation, technological infrastructure, and professional training opportunities.

The economic context should also be considered when interpreting the observed differences in rehabilitation service availability. Although most countries in this study were high-income according to World Bank categories, this classification does not capture differences in rehabilitation financing, workforce distribution, infrastructure, reimbursement models, or the balance between hospital-based and community-based services. The lower availability of early supported discharge, home rehabilitation, and community-based rehabilitation in some countries may therefore reflect not only historical development of rehabilitation medicine, but also differences in how resources are allocated across the stroke rehabilitation pathway. From a patient perspective, such service gaps may affect continuity of care and could plausibly influence functional recovery, participation, quality of life, and long-term dependency, although these outcomes were not measured directly in the present survey.

### Role of PRM physicians

Although PRM physicians are formally responsible for post-stroke rehabilitation in most participating countries, substantial gaps persist in service availability, continuity, and quality. Notably, our findings revealed a marked mismatch between the incidence of stroke and the quantity of the PRM physician workforce in many countries. This imbalance may help explain several current challenges observed across Europe, including: (*i*) the low density of PRM physicians in several countries – often fewer than 1 PRM physician per 100,000 population – which inevitably limits the capacity to deliver timely and comprehensive PRM-led rehabilitation; (*ii*) the limited involvement of PRM specialists during the acute phase, where rehabilitation pathways are frequently initiated and coordinated by other healthcare professions; and (*iii*) the fragmentation of the post-acute and long-term care rehabilitation service, particularly in countries joining ESPRM after 1991. Our findings underscore the urgent need to strengthen the PRM physician workforce. Nevertheless, workforce shortages are likely influenced by the heterogeneity of funding, rehabilitation infrastructure, and specialist training across and within countries. Given the rising burden of stroke across Europe (5), investment in PRM specialist training workforce expansion should be regarded as a strategic priority for strengthening high-quality rehabilitation systems and improving long-term outcomes for stroke survivors.

Although Fig. 2 demonstrates a wide variation in the number of PRM physicians per 100,000 inhabitants, this study was not designed to determine how many PRM physicians in each country work specifically in stroke rehabilitation. This is an important distinction for health service planning, because the total number of PRM physicians in a country does not necessarily reflect the workforce available for stroke rehabilitation. Some PRM physicians may work solely in stroke rehabilitation, whereas others may contribute only partly to stroke care while also covering other rehabilitation populations and services. PRM physicians were almost always involved in sub-acute and long-term rehabilitation services, and few were involved in acute rehabilitation. The data show a considerable range in the number of PRM physicians per capita, with a particularly low number in some countries. The observation that PRM physicians are not always officially responsible for stroke rehabilitation in several countries is a critical concern. This suggests gaps in interprofessional collaboration and coordination, potentially impacting the efficacy of stroke rehabilitation. The observed positive correlation (in most countries) between PRM involvement and the provision of post-acute and long-term rehabilitation suggests that increasing the presence and formal integration of PRM physicians could improve stroke rehabilitation provision (7). However, the present data do not allow a direct comparison between countries with high and low PRM physician density in terms of the actual amount, intensity, or scope of PRM intervention in stroke rehabilitation. Therefore, Fig. 2 should be interpreted as describing the general PRM workforce context rather than the stroke-specific PRM workforce. While a higher density of PRM physicians may create greater potential for PRM involvement in stroke rehabilitation, the actual contribution depends on national workforce distribution, service models, professional roles, and whether PRM physicians are allocated specifically to stroke services.

Nevertheless, the observed variation in PRM physician density has important implications for service organization. Countries with fewer PRM physicians per population may have limited capacity to involve PRM specialists across the full stroke pathway, particularly in acute assessment, early rehabilitation planning, early supported discharge, home rehabilitation, and long-term community-based follow-up. In contrast, countries with a larger PRM workforce may have greater potential to integrate PRM physicians across several points in the stroke care continuum. Future workforce planning should therefore consider not only the overall number of PRM physicians, but also their allocation to stroke rehabilitation, their role in different phases of care, and the extent to which they are formally integrated into multidisciplinary stroke pathways.

### Telerehabilitation

There is broad variation in possible use of telerehabilitation services. Beyond this heterogeneity, telerehabilitation has clear potential to become an integral component of the post-stroke continuum of care, particularly to bridge the gap between inpatient discharge and long-term community-based rehabilitation (22). The COVID-19 crisis has highlighted the possibilities that come with remote healthcare services. In this study, telerehabilitation is reported to be used alongside traditional services more than replacing them, emphasizing its value and the need for better access in some areas. There are significant discrepancies between countries. This may stem from variations in healthcare policies, resource allocation, technological infrastructure, and training opportunities for professionals using telerehabilitation technologies. This all underscores the need for further investigation and consensus regarding sound and structured use of telerehabilitation services (22).

### Stroke registries and quality control

The prevalence of national stroke registries and their varying levels of comprehensiveness (coverage and utilization) highlights the uneven landscape of quality control in stroke care across Europe. While registries are a valuable tool for monitoring quality, their absence or inconsistent application in several countries points to harmonizing data collection, reporting, and quality standards across the field. The use of registry data for quality improvement initiatives should be further strengthened and standardized. On the one hand, as part of stroke care, it should be consistent with accepted standards of stroke registries. On the other hand, it should also be consistent with the field of rehabilitation. The World Health Organization, as part of the Rehabilitation in Health Systems – Guide for Action initiative provides a Rehabilitation Indicator Menu (23). Additionally, standardizing health electronic records for stroke patients is crucial to facilitate big data analysis and enhance the understanding of service availability and effectiveness across different regions. Standardized electronic health records will enable more accurate comparisons, better standardized resource allocation, and improved patient outcomes through data-driven insights.

### Limitations

National delegates from various European countries were asked to report on the organization and delivery of stroke rehabilitation services in their respective countries. This reliance on self-reported data introduces several potential biases, such as incomplete or skewed data due to various interpretations of definitions and availability of listed rehabilitation services. Differences in how delegates interpret and respond to survey questions can lead to inconsistencies in the data. To identify trends in the provision of rehabilitation, participating countries were categorized based on their membership in the ESPRM. However, due to the complex and multifactorial history of Europe and the development of medical rehabilitation, detailed information is not always available. Consequently, this approach may introduce some bias.

Another limitation is that the survey captured expert-reported availability of services rather than direct measures of service capacity, intensity, quality, or patient-level utilization. Therefore, a service reported as available in a country may differ substantially in coverage, accessibility, staffing, and integration within the wider stroke pathway. In addition, where more than 1 respondent provided information for the same country, regional variation may have influenced responses despite attempts to summarize national availability. In addition, the study did not collect detailed economic indicators, rehabilitation expenditure, reimbursement information, or patient-level outcomes; therefore, it was not possible to directly analyse the relationship between economic context, service availability, rehabilitation activity, and patient outcomes.

These limitations may affect the accuracy, reliability, and generalizability of the findings, making it difficult to draw definitive conclusions regarding the overall state of stroke rehabilitation services across Europe. Interpretation is further complicated by the substantial heterogeneity of healthcare systems, governance structures, and rehabilitation service organization across Europe, including regional variation within individual countries. Moreover, the absence of several important European countries, including Germany, Austria, and Finland, limits the completeness of the European overview and may reduce the representativeness of the results.

Taken together, the results of this survey and its limitations converge on the need for more standardized, routinely collected data on stroke rehabilitation across Europe. The observed variation in service availability, regional organization, registry coverage, and quality control, combined with the limitations of expert-reported survey data, indicates that future stroke registries and electronic health records should be a central recommendation arising from this study. Registries should include rehabilitation-relevant indicators across the full stroke pathway, such as access to early supported discharge, inpatient, outpatient, home- and community-based rehabilitation, telerehabilitation, follow-up rehabilitation, functional outcomes, and participation. These indicators should complement established stroke quality frameworks and reflect rehabilitation-specific goals, including functioning, continuity of care, and reintegration into daily life. Standardized electronic health records would enable more consistent data capture across regions and countries, support benchmarking and quality improvement, and allow future European surveys to move beyond expert opinion towards routinely collected data. This would make future assessments of stroke rehabilitation services more robust, comparable, and useful for policy and service planning. To gain a comprehensive understanding, a deep dive into the details of each service is necessary. This includes examining each service’s provision, population, interventions, and assessment methods.

### Conclusion

This study describes the current delivery of the continuum of stroke rehabilitation services across Europe. Addressing these variations requires a multi-faceted approach involving policy changes, resource allocation, and professional training to improve the quality of care and ensure equitable access to services. The findings highlight the urgent need to harmonize stroke rehabilitation service provision, expanding the PRM specialist workforce and establishing common standards and best practices for stroke rehabilitation. This will ultimately improve patient outcomes and reduce the social and economic burden after stroke across Europe.

## Supplementary Material


