# Supplementary material for: SURVEY ON STROKE REHABILITATION IN EUROPE (SUSTAIN-EU) TO EVALUATE EUROPEAN STROKE REHABILITATION SERVICES
Source: J Rehabil Med. 2026 Aug 17;58:44888. doi: 10.2340/jrm.v58.44888 (PMC13488150; doi:10.2340/jrm.v58.44888)

Survey on Stroke Rehabilitation in Europe (SUSTAIN-EU)

to evaluate European stroke rehabilitation services

Stroke SISC ESPRM

What is the aim of the survey?

The overall aim of the survey is to provide an overview and evaluate the contexture of post-stroke rehabilitation service in European countries.

Who is eligible to complete the survey?

The Survey are expected to complete by the national delegates and /or (the national delegates may further distribute the survey to) the key persons in different regions in the country.

Which information is going to be collected?

The survey is collecting data regarding general information on stroke and stroke rehabilitation, telerehabilitation, quality registries and control.

Why should I participate the survey?

The data will serve as a unique recourse to compare and contrast different stroke rehabilitation services delivery systems not only between the countries but also between the different regions within a country. It will enhance the scarce knowledge regarding optimal stroke rehabilitation service delivery in Europe

What should I do, if I can't answer the question?

If you have no answer to the certain question, indicate that no information is available (such as "NA").

Thank you very much for your participation!

The link to the on-line survey: https://forms.gle/3HprkWiw37K5WFTU7

General Information

1.1. Information on person who is filling the form

Name, surname		Click or tap here to enter text.	
Gender		Male ☐	Female ☐
Career level		Senior Consultant ☐	
		Consultant ☐	
		Resident ☐	
Specialization		Click or tap here to enter text.	
E-post address		Click or tap here to enter text.	
Position in ESPRM		Nationtional delegate Yes ☐ No ☐	
Affiliation	Institution	Click or tap here to enter text.	
	City	Click or tap here to enter text.	
	Country	Click or tap here to enter text.	
Representation	Name of the country	Click or tap here to enter text.	
	N of regions in the country		
	Name of the region (if applicable)	Click or tap here to enter text.	
	Population	Click or tap here to enter text.	
Please, describe differences in stroke care of rehabilitation among the different regions?		Click or tap here to enter text.	

1.2. *Number of PRM physicians*

Absolute number	Click or tap here to enter text.
Physicians officially responsible for acute rehabilitation for stroke patients	Click or tap here to enter text.
Physicians responsible for post-acute/long term rehabilitation for stroke rehabilitation	Click or tap here to enter text.

1.3. Stroke

Data should be rate for /100 000 inhabitants			
The estimated annual number of the incident strokes (N/100 000 inhabitants)		Click or tap here to enter text.	Cannot be estimated ☐
The estimated annual number of the incident strokes with rehabilitation needs (N/100 000 inhabitants)		Click or tap here to enter text.	Cannot be estimated ☐
The estimated annual number of the incident transient ischemic attack (TIA) (N/100 000 inhabitants)		Click or tap here to enter text.	Click or tap here to enter text.
The estimated annual number of the incident TIA with rehabilitation needs (N/100 000 inhabitants)		Click or tap here to enter text.	Cannot be estimated ☐
Provided information is based on:	registries	Yes ☐	No ☐
	estimates	Yes ☐	No ☐
	others	Yes ☐	No ☐

			Please, specify: Click or tap here to enter text.
1.4. *Organization of stroke rehabilitation care system*			
In your country, is there an established/ defined plan/program/strategy to address stroke rehabilitation recommendations?		Yes ☐	No ☐
If yes, at what level operates the stroke rehabilitation plan/program/strategy?		National ☐<br>Regional ☐<br>Local ☐<br>Other, please specift Click or tap here to enter text.	
In your country, is there an established/ defined stroke rehabilitation pathway?		Yes ☐	No ☐
Which rehabilitation guidelines are implemented in your country?		World Health Organization's ☐<br>European Stroke Organization's ☐<br>National ☐<br>Others, please specifyClick or tap here to enter text.<br>None ☐	
Does the health administration apply any ¨pay for performance¨ strategy that is based on performance measures for stroke rehabilitation?		Yes ☐	No ☐
Does your country has stratification of stroke – service departments (stroke unit, stroke centre, comprehensive stroke unit) according to Action Plan for stroke in Europe 2018-2030?		Yes ☐	No ☐
If you have a stroke registry what proportion of stroke patients is covered by the registry (%)?		Click or tap here to enter text.	
Which services of the stroke rehabilitation is provided in your country?			
	Rehabilitation in stroke unit		☐
	Rehabilitation in acute ward		☐
	Early supported discharge		☐
	In-patient rehabilitation		☐
	Day rehabilitation		☐
	Home rehabilitation		☐
	Out-patient/ambulatory rehabilitation		☐
	Community based rehabilitation		☐

	Follow-up/intermittent rehabilitation	☐
	Telerehabilitation	☐
	Other, please specify	Click or tap here to enter text.

Definitions of stroke rehabilitation services

**Stroke Unit:**	Organised stroke unit care is a form of care provided in hospital by nurses, doctors and therapists who specialise in looking after stroke patients and work as a co-ordinated team. (1)Organised inpatient (stroke unit) care is a term used to describe the focusing of care for stroke patients in hospital under a multidisciplinary team who specialise in stroke management. (2)
**Rehabilitation for stroke patients in acute wards**	Establishment of a mobile visiting PRM team under the responsibility of a specialist in PRM, while the patient remains in the referring specialist's bed (acute rehabilitation team (ART). • Daily visits to the acute wards by specialists from a standalone PRM facility. • Establishment of facilities in PRM centres to take patients very early to start their PRM programme. (3)
**Early supported discharge**	Team offering rehabilitation in the community that replicates the stroke unit care; this enables earlier home discharge than would be possible if the team was not available (reach out or reach in). (4)
**In-patient rehabilitation**	Goal-oriented multi-professional rehabilitation under the responsibility of a PRM physician in the in-patient rehabilitation facility. (5)
**Day rehabilitation**	Goal-oriented multi-professional rehabilitation under the responsibility of a PRM physician in the out-patient rehabilitation facility. (6)
**Out-patient – ambulatory rehabilitation**	Single rehabilitation services in the out-patient rehabilitation facility. (6)
**Home rehabilitation**	Rehabilitation at home provided by interprofessional teams to replace institutional rehabilitation (7)
**Home based rehabilitation**	Home exercise done by patients themselves following professional instructions to prevent deterioration and promote health through physical activity. (7)
**Community based rehabilitation**	Strategy within general community development for the rehabilitation, equalization of opportunities, poverty reduction and social inclusion of people with disabilities (8)
**Follow-up or intermittent rehabilitation**	Intermittent inpatient rehabilitation services can be used to induce and bolster rehabilitation effects in patients with chronic health conditions, in particular if they are related to psychosocial stress and vocational problems. (9)
**Telerehabilitation**	The delivery of rehabilitation services via information and communication technologies (10)

Telerehabilitation

	Rehabilitation in stroke unit	Rehabilitation in acute ward	Early supported discharge	In-patient rehabilitation	Day rehabilitation	Home rehabilitation	Out-patient/ambulatory rehabilitation	Community based rehabilitation	Follow-up/intermittent rehabilitation	Telerehabilitation	Other specified
**Telerehabilitation is used to:**											
Replace existing services	☐	☐	☐	☐	☐	☐	☐	☐	☐	☐	☐
Complement existing services	☐	☐	☐	☐	☐	☐	☐	☐	☐	☐	☐
**For what purposes telerehabilitation is used in stroke care?**											
Assessment/evaluation of patient	☐	☐	☐	☐	☐	☐	☐	☐	☐	☐	☐
Diagnostics	☐	☐	☐	☐	☐	☐	☐	☐	☐	☐	☐
Setting goals	☐	☐	☐	☐	☐	☐	☐	☐	☐	☐	☐
Treatment/ rehabilitation therapy	☐	☐	☐	☐	☐	☐	☐	☐	☐	☐	☐
Education	☐	☐	☐	☐	☐	☐	☐	☐	☐	☐	☐
Monitoring/follow-up	☐	☐	☐	☐	☐	☐	☐	☐	☐	☐	☐
**In what way telerehabilitation is provided? (11)**											
2-way real-time visits with audio and/or video	☐	☐	☐	☐	☐	☐	☐	☐	☐	☐	☐
Asynchronous e-visits	☐	☐	☐	☐	☐	☐	☐	☐	☐	☐	☐
Virtual check-ins	☐	☐	☐	☐	☐	☐	☐	☐	☐	☐	☐
Remote evaluations of recorded videos or images	☐	☐	☐	☐	☐	☐	☐	☐	☐	☐	☐
Telephone assessment and management services	☐	☐	☐	☐	☐	☐	☐	☐	☐	☐	☐

Stroke rehabilitation registries and quality control

Does your country have National Stroke register	Yes ☒	No ☒
If yes, what proportion of stroke patients is covered by the registry	Click or tap here to enter text.	
Does your country have National Rehabilitation register	Yes ☐	No ☒
If yes, what proportion of stroke patients is covered by the registry	Click or tap here to enter text.	

Is there:	Rehabilitation in stroke unit	Rehabilitation in acute ward	Early supported discharge	In-patient rehabilitation	Day rehabilitation	Home rehabilitation	Out-patient/ambulatory rehabilitation	Community based rehabilitation	Follow-up/intermittent rehabilitation	Telerehabilitation	Other specified
Assessment/evaluation of service delivery quality?	☐	☐	☐	☐	☐	☐	☐	☐	☐	☐	☐
Identification of problems or shortcomings in service delivery?	☐	☐	☐	☐	☐	☐	☐	☐	☐	☐	☐
Designing activities to overcome these deficiencies?	☐	☐	☐	☐	☐	☐	☐	☐	☐	☐	☐
Follow-up monitoring to ensure effectiveness of corrective steps?	☐	☐	☐	☐	☐	☐	☐	☐	☐	☐	☐
Accreditation/certification	☐	☐	☐	☐	☐	☐	☐	☐	☐	☐	☐
Audit	☐	☐	☐	☐	☐	☐	☐	☐	☐	☐	☐
Appointed quality manager	☐	☐	☐	☐	☐	☐	☐	☐	☐	☐	☐
Single intervention explicitly to improve structure/process/outcome quality	☐	☐	☐	☐	☐	☐	☐	☐	☐	☐	☐

REFERENCES

1. Stroke Unit Trialists C. Organised inpatient (stroke unit) care for stroke. Cochrane Database Syst Rev. 2013: CD000197.
2. Stroke Unit Trialists C. Organised inpatient (stroke unit) care for stroke. Cochrane Database Syst Rev. 2002: CD000197.
3. Ward AB, Gutenbrunner C, Damjan H, Giustini A, Delarque A. European Union of Medical Specialists (UEMS) section of Physical & Rehabilitation Medicine: a position paper on physical and rehabilitation medicine in acute settings. J Rehabil Med. 2010; 42: 417-24.
4. Party ISW. National clinical guideline for stroke; 2016.
5. European P, Rehabilitation Medicine Bodies A. White Book on Physical and Rehabilitation Medicine (PRM) in Europe. Chapter 8. The PRM specialty in the healthcare system and society. Eur J Phys Rehabil Med. 2018; 54: 261-78.
6. Gutenbrunner C, Blumenthal M, Geng V, Egen C. Rehabilitation Services Provision and Payment. Am J Phys Med Rehabil. 2017; 96: S35-S40.
7. Mayo NE. Stroke Rehabilitation at Home: Lessons Learned and Ways Forward. Stroke. 2016; 47: 1685-91.
8. World Health Organization U, International Labour Organization & International Disability Development Consortium. Community-based rehabilitation: CBR guidelines.: World Health Organization; 2010.
9. Gutenbrunner C, Bickenbach J, Melvin J, Lains J, Nugraha B. Strengthening health-related rehabilitation services at national levels. J Rehabil Med. 2018; 50: 317-25.
10. Brennan DM, Mawson S, Brownsell S. Telerehabilitation: enabling the remote delivery of healthcare, rehabilitation, and self management. Stud Health Technol Inform. 2009; 145: 231-48.
11. Prvu Bettger J, Resnik LJ. Telerehabilitation in the Age of COVID-19: An Opportunity for Learning Health System Research. Phys Ther. 2020; 100: 1913-6.

	Country	Name, Surname	City	Institution	Position in ESPRM
1	Belgium	Lejeune Thierry	Brussels	Cliniques universitaires Saint-Luc	National delegate of ESPRM
2	Croatia	Darija Granec	Krapinske Toplice	Special hospital for medical rehabilitation Krapinske Toplice	No position in ESPRM
2	Croatia	Sasa Moslavac	Novi Marof	GH Varazdin	No position in ESPRM
3	Czech Republic	Martina Kövári	Prague	Teaching University Hospital Motol	National delegate of ESPRM
3	Czech Republic	Yvona Angerova	Prague	Department of Rehabilitation Medicine, First Faculty of Medicine Charles University and General Teaching Hospital in Prague	National delegate of ESPRM
4	Estonia	Aet Lukmann	Tartu	Tartu University Hospital, University of Tartu	National delegate of ESPRM
5	France	Francois Constant Boyer	Reims	Université Reims Champagne Ardenne	National delegate of ESPRM
6	Greece	Markos Sgantzos	Larissa	Medical School, University of Thessaly	National delegate of ESPRM
7	Hungary	Zoltan Denes	Budapest	National Institute for Medical Rehabilitation	National delegate of ESPRM
7	Hungary	Gabor Fazekas	Budapest	National Institute for Medical Rehabilitation	National delegate of ESPRM
8	Ireland	Paul Carroll	Dublin	National Rehabilitation Hospital, St Vincent's University Hospital, The Royal Hospital Donnybrook, Health Service Executive, Royal College of Physicians of Ireland	National delegate of ESPRM
9	Israel	Iuly Treger	Beer Sheva	Soroka Medical University Center	National delegate of ESPRM
10	Italy	Maria Gabriella Ceravolo	ANCONA	Neurorehabilitation Clinic - Ancona University Hospital , Politecnica delle Marche University	Other
10	Italy	Alessandro Giustini	Arco (Trento)	Ospedale San Pancrazio	Other
11	Latvia	Guna Berzina	Riga	Riga Stradiņš University	Other

12	Luxembourg	Leches Marguerite	Luxembourg	Centre Hospitalier de Luxembourg	National delegate of ESPRM
15	Moldova	Alisa Tabirta	Chisinau	Nicolae Testemitanu State University of Medicine and Pharmacy of the Republic of Moldova.	National delegate of ESPRM
14	Malta	Stephen Zammit	Pieta	Karin Grech Rehabilitation Hospital	National delegate of ESPRM
16	Netherlands	Vera de Vries	Dordrecht	Rijndam	No position in ESPRM
13	North Macedonia	Valentina Koevska	Skopje	University Clinic for Physical Medicine and Rehabilitation, Faculty of Medicine, Ss Cyril and Methodius University	National delegate of ESPRM
17	Norway	Eirik Vikane	Bergen	Haukeland University Hospital	National delegate of ESPRM
18	Poland	Iwona Sarzyńska-Długosz	Warsaw	Institute of Psychiatry and Neurology	No position in ESPRM
19	Portugal	Catarina Aguiar Branco	Feira	Hospital Feira - ULSEDV, Portugal	National delegate of ESPRM
20	Romania	Popa Daiana	Felix Spa	Rehabilitation Hospital Felix Spa	National delegate of ESPRM
21	Russia	Andrei Belkin	Yekaterinburg	Clinical institute of Brain	National delegate of ESPRM
22	Serbia	Milica Lazovic	Belgrade	Institute for Rehabilitation, Belgrade, State University of Novi Pazar, Department of Rehabilitation, Serbia	National delegate of ESPRM
23	Slovakia	Peter Takáč	Košice	Medical Faculty of PJ.J. Šafárik University and L. Pasteur University Hospital in Košice, Slovakia	National delegate of ESPRM
23	Slovakia	Karol Hornáček	Bratislava	Medical Faculty, Slovak healtcare University	National delegate of ESPRM
24	Slovenia	Nataša Bizovičar	Ljubljana	University Rehabilitation Institute Republic of Slovenia	No position in ESPRM
25	Spain	Pia Spottorno Rubio	Madrid	Hospital Universitario La Princesa	No position in ESPRM
26	Sweden	Xiaolei Hu	Umeå	Umeå Univerisity Hospital	National delegate of ESPRM

27	Switzerland	Maximilian Schindler	Geneva	Hirslanden Clinique La Colline	National delegate of ESPRM
28	Turkey	Ayse Kücükdeveci	Ankara	Ankara University	National delegate of ESPRM
29	Ukraine	Volodymyr Golyk	Dnipro	City clinical hospital #4 of Dnipro city council	National delegate of ESPRM
30	United Kingdom	Manoj Sivan	Leeds	Leeds Teaching Hospitals NHS Trust	National delegate of ESPRM

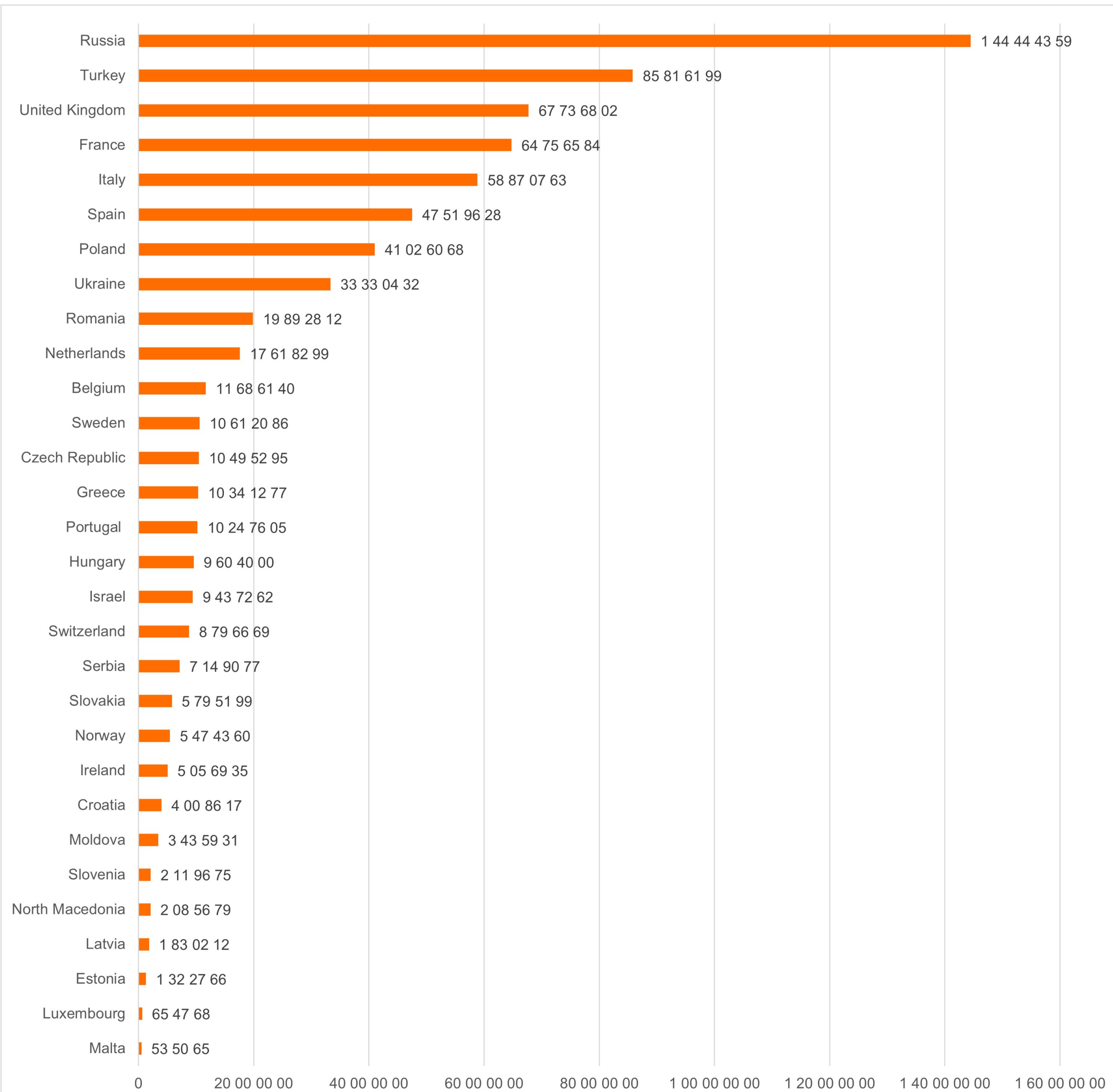

Supplement: Supplementary file 1 [file JRM-58-44888-s1.pdf]
